# The Utility of High-Intensity, Intermittent Exercise Protocols to Induce Fatigue for Screening Purposes in Jump-Landing Sports

**DOI:** 10.5114/jhk/183537

**Published:** 2024-05-17

**Authors:** Stefan Vermeulen, Camilla De Bleecker, Valentien Spanhove, Jan Boone, Tine Willems, Jos Vanrenterghem, Philip Roosen, Roel De Ridder

**Affiliations:** 1 Department of Rehabilitation Sciences, Ghent University, Ghent, Belgium; 2 Department of Rehabilitation Sciences, Catholic University of Leuven, Leuven, Belgium; 3 Department of Movement and Sports Sciences, Ghent University, Ghent, Belgium

**Keywords:** recovery, isokinetic dynamometry, physiology, performance, exertion

## Abstract

Short-term fatigue protocols simulating sports participation are scarce and not well-documented in jump-landing sports. Therefore, this study investigated physiological and physical responses following high-intensity, intermittent exercise protocols (HIIPs) with a standardized level of subjective exhaustion (Borg ≥18/20) and a modified fixed version of five circuits (HIIP-5) for future inclusion in biomechanical screening protocols. Twenty male volleyball and basketball players participated in this study to complete the HIIP and the HIIP-5. Physiological and physical variables were assessed before and up to 30 min after cessation of both protocols. Regarding physiological variables, heart rate values increased (+104 bpm, p < 0.001) and remained elevated up to 30 min (+34 bpm, p < 0.001), and blood lactate levels increased (+17 mmol/l, p < 0.001) compared to baseline. Regarding physical variables, decreased jump height (−4 cm, p = 0.001–0.009) and quadriceps muscle strength (p = 0.001−0.050) were observed up to 30 min compared to baseline. The type of the fatigue protocol did not have an effect on the investigated variables (p > 0.05). To conclude, both the HIIP and the HIIP-5 seem valuable tools to induce acute and long-lasting responses, providing a sufficiently large time window of 30 min within which biomechanical markers of injury can be assessed under fatigued conditions in future risk factor screenings. In practice, the fatigue protocol can be terminated after only five circuits if athletes had not yet been stopped at that point due to exhaustion (Borg ≥18/20).

## Introduction

Fatigue can be defined as an exercise-induced decline in performance and is often considered to be a candidate risk factor for injuries in jump-landing sports such as volleyball and basketball (Lewis, 2018; Vermeulen et al., 2023a; Verschueren et al., 2020; Xu et al., 2023). Therefore, risk factor screenings are often performed when fatigued (Barber-Westin and Noyes, 2017; Benjaminse et al., 2019; Santamaria and Webster, 2010; Verschueren et al., 2020). Whilst full-length match simulations may be most effective in inducing sports-specific fatigue (Bossuyt et al., 2016), they are very time-consuming and thus, there is a need for short duration sports-specific fatigue protocols. These short-lasting protocols still need to be representative by inducing both local and central fatigue effects (Santamaria and Webster, 2010). Moreover, these effects need to last for a sufficient amount of time to be able to perform the screening of the desired outcome variables in a fatigued state (Santamaria and Webster, 2010). The short-term high-intensity SAFT-5 protocol is a soccer specific fatigue protocol which has been validated for inducing fatigue effects for up to 30 min, allowing for a biomechanical screening in a fatigued state (Bossuyt et al., 2016; Smeets et al., 2019). For jump-landing sports, information is currently still lacking about the extent to which fatigue effects can be induced through a similar short-lasting protocol.

Recently, a short-term high-intensity, intermittent exercise protocol (HIIP) was developed for field-sports (Whyte et al., 2018a–c). This protocol seems suitable to represent jump-landing sports since it includes jump activities and has been shown to induce jump-landing control alterations in terms of less hip and knee joint flexion and more proximal compensations (e.g., more trunk flexion, less anterior pelvic tilt) (Whyte et al., 2018a–c). The HIIP includes intermittent, high-intensity bouts of directional changes and jumps until a subjective Borg score ≥18/20 is reached. Notwithstanding the use of the Borg rating, few other means have been documented to objectify fatigue effects or their retention after finishing the protocol. Thus, it is currently unclear whether the HIIP induces acute and long-lasting physiological (e.g., the heart rate and blood lactate concentrations), physical (e.g., jump height and muscle force) and perceived (e.g., the rate of perceived exertion) responses associated with participation in jump-landing sports (Aoki et al., 2017; Bossuyt et al., 2016; Claudino et al., 2017; Stojanović et al., 2018).

Besides that, the HIIP does not terminate after a fixed amount of work (e.g., distance/time), making it unrepresentative for match play and inappropriate for implementation in prospective cohort studies with large sample sizes (Bossuyt et al., 2016). The original HIIP studies showed an average of 6–7 completed circuits (SD = 1.8−2.7) in varsity athletes and Gaelic soccer players (Whyte et al., 2018a–c), and a plateau of physiological variables (e.g., heart rate and Borg scores) was observed at the fifth circuit in the pilot work examining volleyball and basketball players. Therefore, a modified fixed version of five circuits (HIIP-5) could be an easily plannable alternative for the traditional HIIP for future inclusion in prospective studies. However, it is currently unclear whether both the HIIP and the HIIP-5 induce similarly sufficient fatigue effects for up to 30 min.

Therefore, the first purpose of this study was to evaluate the capacity of both the HIIP and the HIIP-5 to induce fatigue and cardiovascular stress by monitoring acute physiological (heart rate and blood lactate levels), physical (jump height and muscle force) and perceived (the rate of perceived exertion for breathlessness and legs) responses. Subsequently, the retention of these responses up to 30 min following protocol cessation was evaluated. This time window would be needed if researchers wish to perform risk factor screenings assessing markers of injury (e.g., muscular, biomechanical) in a fatigued state. The second purpose of this study was to determine whether the circuit-based variant (HIIP-5) could serve as a valid alternative for the original HIIP. In analogy with the responses observed after the SAFT-5 in soccer (Bossuyt et al., 2016), acute and long-lasting responses were also expected to be induced by both the HIIP and the HIIP-5 in jump-landing sports, supporting the use of either of these protocols for risk factor screenings under fatigued conditions.

## Methods

### Participants

This cross-sectional repeated measures cross-over study was registered at ClinicalTrials.gov (ID = NCT04531891), approved by the Ethics Committee of the Ghent University Hospital (approval code: BC-07679, date of approval: 07 August 2020), and written informed consent was obtained from each participant. An a priori sample size of ≥13 players was estimated to observe a reduction in jump performance (i.e., jump height) of 2.8 ± 2.2 cm after fatigue (*power* = 0.80, *α* = 0.004, *d* = 0.61) (Scanlan et al., 2018). To be included in this study, participants had to meet the following criteria: (1) male, (2) playing volleyball or basketball ≥3 times per week, (3) ≥18 years of age, and (4) no history of lower extremity injuries in the past 6 months. Finally, we included 10 volleyball and 10 basketball players (Table 1).

**Table 1:** Participants’ characteristics (mean ± SD).

	Total group (n = 20)	Volleyball (n = 10)	Basketball (n = 10)	Volleyball vs. basketball (*p*-value and Cohen’s *d*)
Age (y)	23.4 ± 4.0	24.2 ± 4.3	22.5 ± 3.7	*p* = 0.351, *d* = 0.43
Height (cm)	186.9 ± 8.0	184.0 ± 4.3	189.9 ± 9.9	*p* = 0.105, *d* = 0.76
Weight (kg)	80.1 ± 10.2	74.9 ± 5.9*	85.4 ± 11.2*	*p* = 0.020, *d* = 1.17
BMI (kg/m^2^)	22.9 ± 2.1	22.1 ± 1.8	23.6 ± 2.2	*p* = 0.104, *d* = 0.77
Hours of sports activity per week (h)	6.8 ± 1.7	7.3 ± 0.8	6.3 ± 2.2	*p* = 0.199, *d* = 0.61
Number of days of sports activity per week (d)	3.2 ± 0.9	3.0 ± 0.0	3.4 ± 1.2	*p* = 0.382, *d* = 0.41
Number of years of sports experience (y)	14.3 ± 4.9	13.9 ± 5.7	14.8 ± 4.2	*p* = 0.676, *d* = 0.19

BMI = Body mass index; cm = centimetre; d = days; h = hours; kg = kilograms; m = metre; y = years. * Statistically significant difference between volleyball and basketball players (p < 0.05).

### Procedures

Players visited the lab twice to complete either the HIIP or the HIIP-5. The order of both sessions was a priori randomly assigned and sessions were separated by more than 48 h. Before each session, players were asked to (1) avoid heavy meals for ≥2 h, (2) refrain from caffeine, energy drinks and smoking at the day of testing, and (3) avoid strenuous exercise for ≥48 h. Each session started with a 10-min warm-up (execution of the fatigue protocol at low speed without inducing any fatigue). To minimize potential accumulation of fatigue, players were instructed to execute one circuit of the fatigue protocol at low speed after the baseline tests and sit quietly for 15 min between the post-fatigue measures.

### Fatigue Protocol

**Figure 1:**
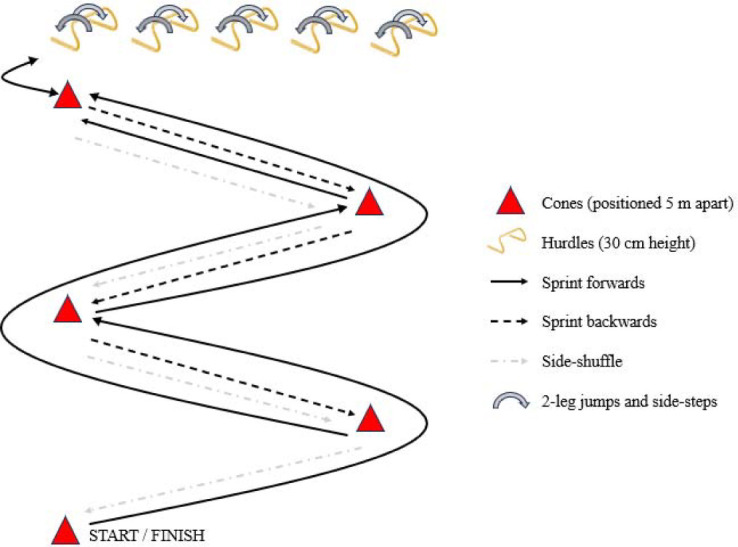
Fatigue protocol.

The HIIP fatigue protocol consisted of intermittent bouts of high-intensity exercise during the execution of functional circuits (i.e., sprints, directional changes, jumps and side-stepping tasks, performed at the highest possible movement speed), interspersed with passive rest periods of 30 s (Whyte et al., 2018a−c). During the circuits, participants were asked to sprint forwards 5 m, cut off at a 90° angle, sprint forwards another 5 m and sprint backwards 5 m. This was repeated until arriving at the hurdles, where 10 jumps and 10 side-steps were performed. Finally, participants side-shuffled around the cones back to the starting point (Figure 1) (Whyte et al., 2018a−c). During the rest periods after each circuit, participants were asked to score their perceived exertion on a Borg scale from 6 (“no exertion at all”) to 20 (“maximal exertion”), indicating the degree to which they felt the body was working (e.g., breathing rate, muscular effort). As such, both the rate of perceived exertion for breathlessness (RPE-B) and legs (RPE-L) were measured (Borg et al., 2010). According to previous HIIP studies (Whyte et al., 2018a−c), the HIIP was terminated when participants achieved a score of ≥18/20 (“very hard”) on the RPE-B and/or the RPE-L scale. This score was suggested to be ‘safe’ while still eliciting muscular fatigue (Abergel et al., 2021). The HIIP-5 was terminated when a fixed number of five circuits was completed. Failure to complete five circuits during the HIIP-5 resulted in exclusion of the data from statistical analysis.

### Data Collection

The timing of examining the outcome variables before, during and after the fatigue protocols is presented in Table 2. For physiological variables, the heart rate (HR) was monitored with a Polar system (Polar, Electro, Finland). The HR obtained at the end of the protocols can be considered a marker of cardiovascular stress during the protocol (Stojanović et al., 2018), whereas the recovery of the HR in the timeframe following exercise can reflect the recovery from the homeostatic stress induced by the exercise (Mann et al., 2014). Blood lactate levels were registered using a Lactate Pro 2 Meter (Samcon, Belgium). First, capillary blood samples were drawn from the fingertip after cleaning and drying the finger from excessive sweat (Hertogh et al., 2005). Thereafter, blood lactate levels were determined from these samples and peak values were compared to baseline in order to establish the metabolic perturbation and reflect the metabolic acidosis induced by the protocol (Goodwin et al., 2007; Marcinek et al., 2010).

**Table 2:** Timing of measurements during the fatigue protocols.

	PRE	DURING*	POST							
**TIME (min)**			0	2	5	10	15	20	25	30
Heart rate										
Blood lactate concentration										
Jump height										
Quadriceps muscle strength at 120°/s and 300°/s										
Circuit time										
RPE-B, RPE-L										

RPE-B = Rate of perceived exertion for breathlessness; RPE-L = Rate of perceived exertion for legs. *After each circuit during the fatigue protocols.

For the physical characteristics, jump height and maximal muscle strength of the quadriceps were determined to assess muscular fatigue (Bossuyt et al., 2016). Jump height was measured using Optojump Photo-electric cells (Microgate Optojump Next, The Netherlands) in which the average height of three maximal counter-movement jumps was determined (Claudino et al., 2017). Therefore, participants were instructed to keep their arms akimbo and to jump as high as possible without pulling their knees up during the flight. Second, concentric (CONC) quadriceps muscle strength was assessed using isokinetic dynamometry (IKD, Biodex System 3, Shirley, NY). Therefore, players performed five maximal knee extensions at moderate (joint angular) velocity (120°/s) and five at high velocity (300°/s). These velocities were chosen since a previous study demonstrated high knee angular velocities (>300°/s) during stop-jump tasks, which have been associated with increased knee injury risk (Bisseling et al., 2008). During the IKD assessments, participants flexed their knee with sub-maximal effort to avoid hamstring muscle fatigue. Strength evaluations were always performed on the participants’ leading leg when performing a stop-jump task, since the pilot work showed higher impact forces for this leg. Two practice trials were allowed before the baseline physical tests. Additionally, circuit time during the protocols was registered with infrared timing gates (Microgate, The Netherlands).

### Data Analysis

Raw joint torque data were extracted from the IKD and imported in Matlab (MathWorks, Inc., Natick, MA, USA) for individual curve analysis using IKD1D (www.ikd1d.org). During this process, only gravity-corrected torque-angle data measured within 10% velocity tolerance at 120°/s or 300°/s were selected. Afterwards, a fourth order polynomial fit curve was calculated for ≥3 valid trials. The common divider for the knee joint range was eventually calculated for all participants, resulting in torque data analysed between 95° and 35° of knee flexion for the IKD at 120°/s, and between 85° and 45° of knee flexion for the IKD at 300°/s. These ranges appeared to be similar to those observed during the greatest part of the landing phase of the stop-jump task (Vermeulen et al., 2023b). All torque data were normalized to body mass (Nm/kg).

### Statistical Analysis

The statistical analysis of the discrete variables was performed in IBM SPSS statistics 26. Isokinetic torque-angle profiles were compared using Statistical Parametric Mapping (SPM, www.spm1d.org) in Matlab (MathWorks, Inc., Natick, MA, USA) (Pieters et al., 2022). For all discrete variables, normality was first checked with the Shapiro-Wilk test and corresponding normality plots. Two-way repeated measures ANOVA was performed for all normally distributed outcome variables (or the Friedman test for non-normally distributed variables) to investigate any interaction effect (protocol*fatigue), or any main effect of the protocol (HIIP vs. HIIP-5) or fatigue (PRE vs. POST protocol). Post-hoc paired sample *t*-tests (or Wilcoxon signed-rank tests) with Bonferroni correction were applied when a statistically significant effect was found (*α* = 0.05). Partial eta squared (*np^2^*), which is the effect size measuring the proportion of the total variance in the dependent variable (i.e., outcome variable) that is partially explained by the independent variable (i.e., protocol, fatigue or interaction of protocol*fatigue) (Richardson, 2011), was calculated for the omnibus ANOVA and classified into small (0.01−0.06), medium (0.06−0.14) and large effects (>0.14). For the post-hoc tests, Cohen’s *d* effect sizes, which measure the magnitude of the differences, were calculated and classified as small (0.20−0.50), medium (0.50−0.80) and large (>0.80) (Sullivan and Feinn, 2012).

## Results

### Fatigue Protocol Characteristics

The data of the fatigue protocol characteristics met the assumptions of normal distribution, except for the number of completed circuits and protocol completion time. Participants completed a median number of four circuits during the HIIP (range = 1−23; <5 circuits: n = 12, 5 circuits: n = 3, >5 circuits: n = 5). Protocol completion time did not significantly differ between both protocols (HIIP: median = 4.6 min, range = 0.7−27.8; HIIP-5: median = 5.5 min, range = 5.1−6.6; *p* = 0.296). Circuit time significantly increased from the first to the final lap for both protocols (HIIP: +3.8 ± 3.0 s, *p* < 0.001, *d* = 1.28; HIIP-5: +4.5 ± 3.8 s, *p* < 0.001, *d* = 1.18). Since one volleyball player was not able to complete the HIIP-5, data from 19 vs. 20 players were included for the HIIP-5 and the HIIP in the analysis, respectively.

### Responses Following the Fatigue Protocols

**Figure 2:**
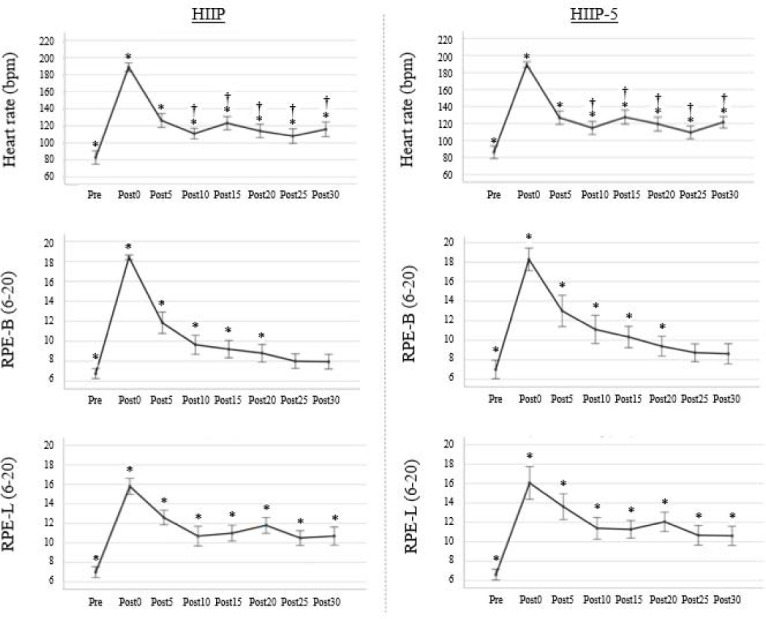
Heart rate and perceived exertion before and after the HIIP and HIIP-5 protocols (mean and 95% CI). * Statistically significant difference between pre- and post-fatigue conditions (p < 0.05). † Statistically significant difference between the HIIP and the HIIP-5 (p < 0.05)

**Figure 3:**
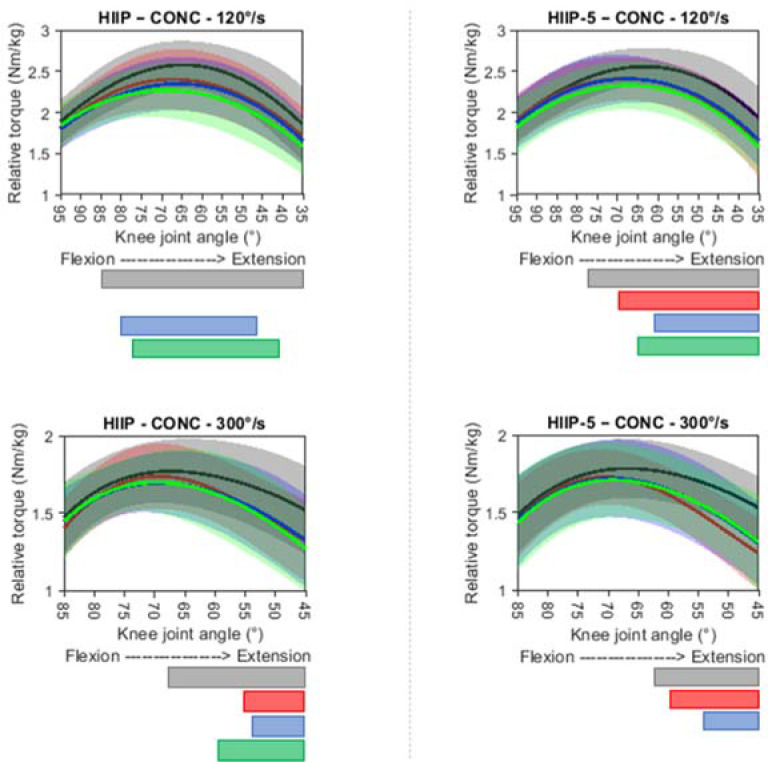
Quadriceps concentric torque-angle profiles at 120°/s and 300°/s before and after the HIIP and HIIP- 5 protocols, presented as mean and standard deviation clouds. *PRE = black; POST0 = red; POST15 = blue; POST30 = green. The transparent areas under the curves indicate those knee joint angles that were significantly affected by fatigue (p < 0.05; effect of fatigue = grey bar; POST0 vs. PRE = red bar; POST15 vs. PRE = blue bar; POST30 vs. PRE = green bar)*.

The blood lactate data of one participant were withdrawn from the statistical analysis due to an experimental error during data collection. The data of all responses following the protocols fulfilled the assumptions of normal distribution. Responses following the protocols are presented in Figures 2 and 3 and Table 3, and post-hoc statistics are shown in Table 4. For the total group, no significant interaction and protocol main effects were found for all variables, except for the protocol on the HR (*p* = 0.005, *np^2^* = 0.41) with significantly higher HRs 10−30 min after the HIIP-5 compared to the HIIP (*p* = 0.003−0.024, *d* = 0.47−0.84). A significant main effect of fatigue was found for all variables (HR: *p* < 0.001, *np^2^* = 0.95; blood lactate: *p* < 0.001, *np^2^* = 0.95; jump height: *p* < 0.001, *np^2^* = 0.59; quadriceps muscle strength at 120°/s: *p* < 0.001, *np^2^* = 0.60, quadriceps muscle strength at 300°/s: *p* = 0.005, *np^2^* = 0.55; RPE-B: *p* < 0.001, *np^2^* = 0.91; RPE-L: *p* < 0.001, *np^2^* = 0.81). Post-hoc comparisons showed that the HR and RPE-L scores significantly increased and remained elevated up to 30 min after both protocols. RPE-B scores also significantly increased, but only up to 20 min after both protocols. Peak blood lactate levels significantly increased after both protocols. Jump height and quadriceps muscle strength at 120°/s and 300°/s significantly decreased up to 30 min after both protocols, except for quadriceps strength at 120°/s immediately after the HIIP and for quadriceps strength at 300°/s at 30 min after the HIIP-5.

**Table 3:** Physiological, physical and perceived responses following the HIIP and HIIP-5 protocols (mean ± SD).

	Fatigue protocol	PRE	POST0	POST5	POST10	POST15	POST20	POST25	POST30
Heart rate (bpm)	HIIP	82.8 ± 15.8*	188.7 ± 10.0*	126.1 ± 16.5*	110.8 ± 12.6*^†^	123.1 ± 15.6*^†^	113.9 ± 16.0*^†^	107.9 ± 17.3*^†^	115.8 ± 17.2*^†^
	HIIP-5	86.3 ± 14.8*	189.1 ± 6.9*	126.8 ± 15.8*	114.8 ± 15.4*^†^	127.5 ± 16.6*^†^	119.4 ± 16.4*^†^	109.6 ± 15.5*^†^	121.5 ± 13.4*^†^
Blood lactate levels (mmol/l)	HIIP	2.4 ± 1.2*	18.7 ± 5.4*						
	HIIP-5	2.5 ± 1.0*	20.2 ± 4.3*						
Jump height (cm)	HIIP	37.7 ± 5.4*	35.3 ± 5.3*			35.3 ± 6.1*			33.6 ± 5.8*
	HIIP-5	37.4 ± 5.8*	34.3 ± 5.4*			34.3 ± 5.7*			33.6 ± 6.6*
RPE-B (6−20)	HIIP	6.8 ± 1.1*	18.4 ± 0.5*	11.9 ± 2.3*	9.7 ± 2.1*	9.2 ± 1.9*	8.8 ± 1.9*	8.0 ± 1.6	8.0 ± 1.6
	HIIP-5	7.0 ± 1.9*	18.3 ± 2.3*	13.0 ± 3.2*	11.1 ± 2.9*	10.3 ± 2.2*	9.4 ± 2.0*	8.7 ± 1.8	8.6 ± 2.1
RPE-L (6−20)	HIIP	7.0 ± 1.2*	15.8 ± 1.8*	12.6 ± 1.6*	10.7 ± 2.2*	11.0 ± 1.8*	11.8 ± 1.7*	10.5 ± 1.6*	10.7 ± 2.0*
	HIIP-5	6.6 ± 1.1*	16.1 ± 3.4*	13.6 ± 2.7*	11.4 ± 2.3*	11.3 ± 1.8*	12.1 ± 2.0*	10.7 ± 2.0*	10.6 ± 2.0*

bpm = beats per minute; cm = centimetre; HIIP = High-intensity, intermittent exercise protocol; mmol/l = millimole per litre; RPE-B = Rate of perceived exertion for breathlessness; RPE-L = Rate of perceived exertion for legs. * Statistically significant difference between pre- and post-fatigue conditions (p < 0.05). † Statistically significant difference between the HIIP and HIIP-5 protocols (p < 0.05).

**Table 4:** Post-hoc paired comparisons of fatigue main effects (PRE vs. POST) for physiological, physical and perceived responses following the HIIP and HIIP-5 protocols.

	Fatigue protocol	POST0 vs. PRE	POST5 vs. PRE	POST10 vs. PRE	POST15 vs. PRE	POST20 vs. PRE	POST25 vs. PRE	POST30 vs. PRE
Heart rate	HIIP	*p* < 0.001 *d* = 5.93	*p* < 0.001 *d* = 2.82	*p* < 0.001 *d* = 1.94	*p* < 0.001 *d* = 2.20	*p* < 0.001 *d* = 1.94	*p* < 0.001 *d* = 1.53	*p* < 0.001 *d* = 1.90
	HIIP-5	*p* < 0.001 *d* = 6.23	*p* < 0.001 *d* = 2.74	*p* < 0.001 *d* = 2.18	*p* < 0.001 *d* = 2.76	*p* < 0.001 *d* = 2.64	*p* < 0.001 *d* = 1.95	*p* < 0.001 *d* = 2.87
Blood lactate levels	HIIP	*p* < 0.001 *d* = 3.21						
	HIIP-5	*p* < 0.001 *d* = 4.05						
Jump height	HIIP	*p* = 0.009 *d* = 0.83			*p* < 0.001 *d* = 1.62			*p* < 0.001 *d* = 3.44
	HIIP-5	*p* = 0.002 *d* = 1.04			*p* < 0.001 *d* = 1.71			*p* < 0.001 *d* = 2.15
Quadriceps muscle strength at 120°/s	HIIP	*p* > 0.05 *d* = 3.32			*p* < 0.001 *d* = 4.50			*p* < 0.001 *d* = 2.80
	HIIP-5	*p* < 0.001 *d* = 1.38			*p* < 0.001 *d* = 1.80			*p* < 0.001 *d* = 2.62
Quadriceps muscle strength at 300°/s	HIIP	*p* = 0.003 *d* = 1.43			*p* = 0.003 *d* = 2.01			*p* < 0.001 *d* = 1.61
	HIIP-5	*p* < 0.001 *d* = 1.26			*p* = 0.003 *d* = 1.60			*p* > 0.05 *d* = 1.91
RPE-B	HIIP	*p* < 0.001 *d* = 9.50	*p* < 0.001 *d* = 2.03	*p* < 0.001 *d* = 1.37	*p* < 0.001 *d* = 1.31	*p* = 0.019 *d* = 0.91	*p* = 0.184 *d* = 0.68	*p* = 0.234 *d* = 0.66
	HIIP-5	*p* < 0.001 *d* = 4.28	*p* < 0.001 *d* = 2.03	*p* < 0.001 *d* = 1.78	*p* < 0.001 *d* = 1.41	*p* = 0.005 *d* = 1.16	*p* = 0.153 *d* = 0.81	*p* = 0.334 *d* = 0.72
RPE-L	HIIP	*p* < 0.001 *d* = 4.21	*p* < 0.001 *d* = 2.34	*p* < 0.001 *d* = 1.63	*p* < 0.001 *d* = 1.88	*p* < 0.001 *d* = 2.04	*p* < 0.001 *d* = 1.64	*p* < 0.001 *d* = 1.68
	HIIP-5	*p* < 0.001 *d* = 2.87	*p* < 0.001 *d* = 2.57	*p* < 0.001 *d* = 1.89	*p* < 0.001 *d* = 2.14	*p* < 0.001 *d* = 2.71	*p* < 0.001 *d* = 1.79	*p* < 0.001 *d* = 1.71

HIIP = High-intensity, intermittent exercise protocol; RPE-B = Rate of perceived exertion for breathlessness; RPE-L = Rate of perceived exertion for legs. p-values and effect sizes (Cohen’s d) are reported for each comparison. Significant differences are highlighted in bold (p < 0.05).

## Discussion

### Synthesis of the Results

To the authors’ knowledge, this is the first study that investigates the utility and validity of sports-specific, high-intensity, intermittent fatigue protocols in jump-landing sports. Our study showed that both HIIP versions induced fatigue and cardiovascular stress, as several significant main effects for fatigue were found. Moreover, the HIIP-5 seems to be a suitable alternative for the HIIP, as few to no significant main effects for the protocol were found.

This study observed acute and long-term responses for physiological, physical and perceived variables after both HIIP versions in volleyball and basketball players. Regarding physiological responses, the HR increased during the protocols up to values that were close to maximal, which reflects profound cardiovascular stress induced by the two protocols. Similar responses were observed in previous HIIP studies (Whyte et al., 2018a−c), and during volleyball and basketball game play, with average physiological intensities above 60−80% of the maximal HR (Rodríguez-Marroyo et al., 2014; Stojanović et al., 2018). Furthermore, the HR remained elevated above resting values for up to 30 min after the protocol completion. This indicates that both fatigue protocols induce extensive homeostatic stress that needs to be restored following exercise (e.g., increased core temperature, removal of lactate, restoration of phosphocreatine) and that a certain timeframe is needed to recover the resting autonomic tone (i.e., activation of parasympathetic activity and withdrawal of sympathetic activity) (Mann et al., 2014). Higher HR values were monitored 10−30 min after the HIIP-5 compared to the HIIP, indicating slower recovery from homeostatic stress after the HIIP-5 in this sample. Blood lactate concentrations increased on average to 19−20 mmol/l after both fatigue protocols, reflecting the strong metabolic perturbation induced by the protocols. It has been shown that an increase in blood lactate concentration is strongly related to the increase in protons (H+) and thus, also to the occurrence of metabolic acidosis (Marcinek et al., 2010). This metabolic acidosis strongly contributes to the occurrence of muscle (peripheral) fatigue (Fitts, 2016). As such, the significant increase in blood lactate concentrations, found in the present study, might be a reflection of this phenomenon. Small increases in blood lactate concentrations have also been demonstrated during volleyball and basketball games (Mroczek et al., 2011; Stojanović et al., 2018), showing that these sports are indeed characterized by high-intensity actions causing metabolic perturbations.

Regarding physical responses, jump height decreased until 30 min after both protocols, which is consistent with the effects observed after the SAFT-5 (Bossuyt et al., 2016). Decreases in jump height have been already demonstrated in volleyball and basketball players when fatigued (Brazo-Sayavera et al., 2017; Lesinski et al., 2016; Scanlan et al., 2018). Quadriceps muscle strength also decreased until 30 min following both protocols, which has previously been associated with decreased jump height (Scanlan et al., 2018). No differences were observed for quadriceps muscle strength at moderate velocity immediately after the HIIP and at high velocity 30 min after the HIIP-5 compared to baseline. However, there was a trend towards a decreased performance at these points (large effect size). Moreover, fast-twitch fibres are more easily fatigued and less quickly recovered than slow-twitch fibres following short-term, high-intensity exercise (Lievens et al., 2020). Therefore, acute and long-term performance decline could be observed after both fatigue protocols.

Regarding perceived responses, RPE-scores at protocol completion were similar for both the HIIP and the HIIP-5, being consistent with previous studies implementing the HIIP (Whyte et al., 2018a−c). Despite acute responses being found, RPE-B scores already returned to baseline values 20 min after both protocols (medium to large effect size). Researchers and coaches need to be aware that objective, physiological and physical responses may still be present when subjective feelings of tiredness appear to already have normalized. Although no long-lasting (perceived) responses were demonstrated in our study, similar RPE-scores at 30 min have been observed after volleyball or basketball training and/or a match (Aoki et al., 2017; Rodríguez-Marroyo et al., 2014).

## Limitations

A large proportion of players was not able to perform five circuits during the HIIP, which is in contrast with previous HIIP studies (Whyte et al., 2018a−c). This can be attributed to lower physical fitness levels since data gathering was performed on average one month into a forced sport stop of the COVID-19 pandemic, which may have resulted in a partial or complete loss of training-induced morphological and physiological adaptations (Wezenbeek et al., 2022). Thus, the HIIP-5 seemed to only be more time-efficient in a small number of ‘fitter’ players (n = 5) who could complete >5 circuits during the HIIP. The question then arises whether the HIIP-5 would also induce sufficient fatigue in this subset of players. In exploration, large effect sizes for reduced jump performance were also observed in these players for up to 30 min after the HIIP-5 (*d* = 2.41−4.79). This suggests that the HIIP-5 may also have clinically important fatigue effects in this subgroup and further supports the hypothesis that this can be a suitable alternative for the HIIP, with the main benefit of being more easily plannable for risk factor screenings in future prospective studies. Future studies could repeat this study on larger numbers of these ‘fitter’ populations to further confirm these preliminary results.

## Practical Implications

The results of this study showed acute and long-term responses up to 30 min after both the traditional HIIP and the new short-lasting HIIP-5. This time window allows coaches, supporting medical staff or researchers to assess fatigue-induced injury risk factors, for example in pre-season screenings. The protocol has the advantage of being easily plannable as it can be terminated after only five circuits if athletes had not yet been stopped at that point due to exhaustion. Therefore, it is recommended that the HIIP-5 is included in pre-season risk factor screenings as tool for inducing fatigue applicable to volleyball and basketball. Recently, the HIIP-5 was implemented in biomechanical research examining the effects of fatigue on patellar tendon loading during stop-jumps in volleyball (Vermeulen et al., 2023b). Protective strategies, including stiff lower extremity landings (i.e., less hip, knee and ankle (dorsi-)flexion) and proximal compensations (i.e., more pelvis-trunk flexion), were observed to reduce patellar tendon loading in the fatigued state. Future prospective studies should, therefore, investigate whether players are more prone to develop patellar tendinopathy if they experience high eccentric patellar tendon loads in the non-fatigued state and/or continue with eccentrically loading the tendon after fatigue (Vermeulen et al., 2023b). From an exercise physiological point of view, both high-intensity, intermittent exercise protocols induced strong homeostatic stress and metabolic acidosis. Whilst not the goal of this study, coaches may, therefore, wish to implement such protocols in endurance training when their physiological target is to increase anaerobic capacity (Rago et al., 2022). In this context, the HIIP can serve as a tool to gradually and ‘safely’ improve the athlete’s capacity to withstand anaerobic exercise for longer periods of time (Abergel et al., 2021). In their progression, coaches should always encourage appropriate strategies (e.g., nutrition, rehydration, cold-water immersion) to improve recovery and counteract the observed prolonged fatigue responses following this type of training (Haller et al., 2022).

## Conclusions

This study showed a sufficiently large time window of 30 min after both the traditional HIIP and the novel HIIP-5, within which athletic performance is reduced and markers of injury (e.g., muscular, biomechanical) can be assessed under fatigued conditions. Moreover, the protocol can be terminated after only five circuits if athletes had not yet been stopped at that point due to exhaustion (Borg ≥18/20).
